# The Effectiveness of Laser Applications and Photodynamic Therapy on Relevant Periodontal Pathogens (*Aggregatibacter actinomycetemcomitans*) Associated with Immunomodulating Anti-rheumatic Drugs

**DOI:** 10.3390/bioengineering10010061

**Published:** 2023-01-04

**Authors:** Maria-Alexandra Martu, Ionut Luchian, Mihai Mares, Sorina Solomon, Oana Ciurcanu, Vlad Danila, Elena Rezus, Liliana Foia

**Affiliations:** 1 Department of Periodontology, Grigore T. Popa University of Medicine and Pharmacy Iasi, Str. Universitatii No. 16, 700115 Iasi, Romania; 2 Department of Public Health, University of Life Sciences Iasi, Str. Mihail Sadoveanu No. 3, 700490 Iasi, Romania; 3 Department of Dento-Alveolar Surgery, Grigore T. Popa University of Medicine and Pharmacy Iasi, Str. Universitatii No. 16, 700115 Iasi, Romania; 4 Department of Rheumatology, Grigore T. Popa University of Medicine and Pharmacy Iasi, Str. Universitatii No. 16, 700115 Iasi, Romania; 5 Department of Biochemistry, Grigore T. Popa University of Medicine and Pharmacy Iasi, Str. Universitatii No. 16, 700115 Iasi, Romania

**Keywords:** photodynamic disinfection, laser, periodontitis, bacteria, immunomodulators, DMARDS, autoimmune disease, rheumatoid arthritis

## Abstract

Considering the current context of the increasing resistance of bacterial species to antibiotics and other antimicrobial agents, a major objective is to develop other antimicrobial approaches, which would be able to inactivate pathogens with considerable effectiveness. Two such methods are photodynamic disinfection therapy and laser irradiation. In view of the immunocompromised status of some patients under immunosuppressive therapy and potential drug interactions that can be established between systemic antimicrobial agents, the research of local, minimally invasive methods of inactivating periodontal pathogens in the context of these systemic therapies with modifying drugs of the immune response is justified. This in vitro study evaluated the antimicrobial action of a diode laser, wavelength 940 nm, and photodisinfection therapy at 670 nm (photosensitizer, 3,7 dimethyl phenothiazine chloride) on a type strain of *Aggregatibacter actinomycetemcomitans*, a known periodontal pathogen, in the presence and absence of active substances used in autoimmune disease therapy (Etanercept, Infliximab, Metothrexate). The association of a conventional antirheumatic drug with anti-TNF-α therapy determined a significantly greater inhibition of the strain of *A. actinomycetemcomitans* compared to monotherapy, in vitro. Photodisinfection caused a significant reduction in bacterial burden after a 30 s exposure in vitro, regardless of the pharmaceutical associations of biological and conventional disease-modifying antirheumatic drugs (DMARDs). Irradiation with a diode laser for 30 s at a power of 5 W caused a greater reduction compared to irradiation with 1 W. The application of laser and photodisinfection induced a significant reduction in *Aggregatibacter actinomycetemcomitans* in vitro and could be considered important adjunctive measures for the eradication of this oral pathogen in the context of immunomodulating therapy.

## 1. Introduction

Periodontal disease is a chronic inflammation of the deep periodontal tissues caused by specific bacteria that adhere and grow on the surfaces of the teeth and that can also generate pathologies of the dental pulp, and, thus, is involved in numerous systemic diseases, including pneumonia, cardiovascular diseases, renal disease, Alzheimer, autoimmune diseases, cancer, etc. [1].

Considering the similarities between aspects of the pathogenesis of periodontal disease and certain autoimmune diseases, such as rheumatoid arthritis, it is of interest that researchers in the field of periodontology are aware of the therapeutic options available [2].

Currently, treatment of periodontitis consists of classic non-surgical and surgical treatments, and also various types of non-conventional therapies, such as physical stimulation (thermal treatment or ultrasound), the application of chemical and antibiotic agents and photostimulation (laser/LED treatment). Regarding phototherapy, it has been reported to accelerate wound healing and reduce post-operative pain and discomfort when using low-level laser radiation (LLLT) [3].

*Aggregatibacter actinomycetemcomitans* IgA and IgE levels are significantly higher in the serum and saliva of periodontitis patients [4]. Moreover, a study observed an increased in vitro resistance of *A. actinomycetemcomitans* to amoxicillin, azithromycin and metronidazole, some of the most frequently prescribed antibiotics in periodontitis patients [5]. 

Considering the current context of the evolution of bacterial species’ resistance to antibiotics and other antimicrobial agents such as triclosan and chlorhexidine, a major objective is to develop other antimicrobial approaches able to inactivate pathogens with considerable effectiveness. A possible option for the local eradication of bacteria is their photodynamic inactivation. By this method, the bacteria are incubated with a photosensitizer and are subsequently irradiated with light of a suitable wavelength. Exposure of the photosensitizer to the light treatment leads to the generation of reactive oxygen species that kill the bacteria through a so-called “oxidative explosion” phenomenon. There are numerous studies in the literature showing promising results for photodynamic disinfection therapy (PDT) with various light sources ranging from blue to infrared against the main pathogens of periodontal disease [6,7,8].

Alternatively, irradiation with a diode-type laser is a similar method, but it acts through other biochemical and biophysical mechanisms and does not use a photosensitizing agent, but instead produces the intended therapeutic effect through the nature of the interactions established between the light radiation and the periodontopathogenic bacteria as well as the host tissue [9,10].

Low-level laser radiation therapy (LLLT) supports the production of an increased amount of adenosine triphosphate at the mitochondrial level, thus facilitating the proliferation of fibroblasts, the release of growth factors and the synthesis of collagen [11,12]. At the same time, in vitro and animal studies have revealed that this therapy suppresses inflammation in the periodontal tissue by modulating the local immune response and reducing the production and release of certain pro-inflammatory cytokines, such as the tumor necrosis alpha factor (TNF-α), interleukin-1β (IL-1β) and prostaglandin E2. In addition, it has been found to improve local microcirculation through angiogenesis and vasodilation, thus alleviating tissue edema and inflammation [13].

Although there are recent studies in the literature analyzing the effect of laser therapy and PDT in periodontal disease, the eradication mechanisms of the involved bacteria are insufficiently explored (Table 1). 

Moreover, no study to date has analyzed the effect of diode laser therapy and that of photodynamic treatment on a periodontopathogen in the context of anti-TNF-α immunomodulatory therapy. Considering the immunocompromised status of some patients undergoing immunosuppressive therapy and the potential drug interactions that can be established between the antimicrobial agents applied at a systemic level, the research of local, minimally invasive methods of inactivating periodontal pathogens in the context of these systemic therapies with modifying drugs of the immune response such as anti-TNF-α is justified.

Considering the multitude of therapies used in the treatment of autoimmune diseases such as rheumatoid arthritis (RA), ankylosing spondylitis, psoriasis, Crohn’s disease and others, that have the role of modulating the host’s response, the aim of the current study is to investigate to what extent they may influence the inactivation capacity of certain adjuvant therapies used in periodontal treatment on a critical periodontopathogen such as *Aggregatibacter actinomycetemcomitans*.

## 2. Materials and Methods

This in vitro study evaluated the antimicrobial effectiveness of the laser radiation emitted by a diode-type laser device (Epic X, Biolase, Foothill Ranch, CA, USA), wavelength 940 nm, continuous mode, 300 μm insert, 9 mm, non-initialized, and also a photodisinfection device (Helbo^®^ Photodynamic Systems GmbH & Co KG, Senden, Germany), 670 nm, 75 mW/cm^2^, light spot 0.06 cm in diameter. For the photodisinfection therapy, we used HELBO Blue photosensitizer^®^, a liquid containing methylene blue (3,7 dimethyl phenothiazine chloride), which has an absorbance maximum at 670 nm. These minimally invasive therapies were applied to a type strain of *Aggregatibacter actinomycetemcomitans* (DSM-8324) in the presence or absence of active substances used in the therapy of rheumatic pathology.

For this purpose, microplates with 96 wells (Deltalab, Rubi Barcelona, Spain) were used, in which 100 μL of the dilutions of the active substances prepared in advance were pipetted, using as a vehicle a brain-heart infusion broth (Bio-Rad, Hercules, CA, USA) supplemented with 20% human blood serum inactivated at 56 °C for 30 min.

The following active substances were tested, in concentrations equivalent to those reached in the plasma of subjects treated with the following dosage regimen:

- Etanercept 2.5 μg/mL (E)
- Infliximab 50 μg/mL (I)
- Metotrexat 2.5 μg/mL (M)
- Etanercept 2.5 μg/mL + Metotrexat 2.5 μg/mL (E + M)
- Infliximab 50 μg/mL + Metotrexat 2.5 μg/mL (I + M)

Next, 100 μL of bacterial inoculum (10^5^ CFU/mL) prepared extemporaneously in the same type of liquid medium, using a 24 h culture on Tryptone Soya Agar (Bio-Rad, Hercules, CA, USA) were pipetted into each well.

**Figure 1:**
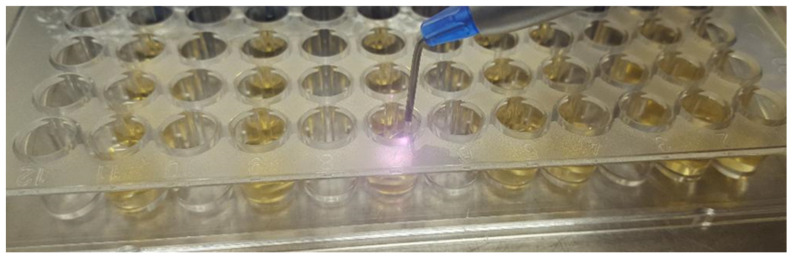
Application of diode laser treatment to *A. actinomycetemcommitans*.

**Figure 2:**
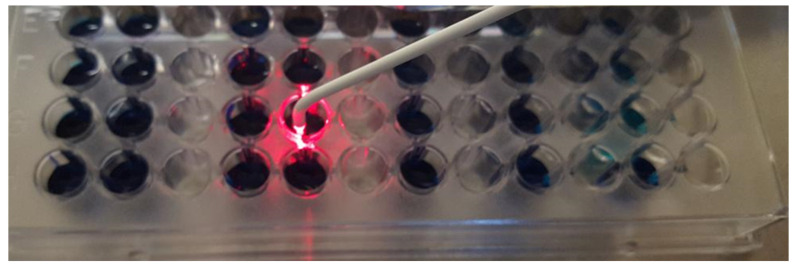
Application of photodisinfection treatment to *A. actinomycetemcommitans*.

In parallel, control wells were also prepared (positive-CP, respectively negative-CN) to evaluate the bacterial growth respective to the sterility of the growth medium. Some of the wells were treated with a Diode type laser (two power regimes: 1 W and 5 W respectively) and with photodisinfection (Figure 1 and Figure 2). Each well was treated for 30 s.

The microplates were then immediately placed in the incubator at 36 °C, ensuring microaerophilic conditions with the help of GENbox anaer sachets (bioMerieux, Craponne, France). After 48 h of incubation, in order to evaluate the degree of bacterial growth, the absorbance of each well was read at a wavelength of 492 nm, with the help of an MR-96 spectrophotometer (Mindray, Shenzhen, China), with the exception of the wells pretreated with photoactivator, in which the interpretation was made by seeding a volume of 10 μL on solid medium and re-incubating (in the case of the positive control, serial dilutions were practiced for the exact determination of the bacterial load existing at the time of the laser treatment in the wells containing photoactivator).

The reduction in turbidity in the wells seeded with the bacterial inoculum and implicitly of the absorbance of their content, correlates with the inhibition of microbial growth. The interpretation was made by comparison with the absorbance of the positive control wells, considered the standard of microbial growth (100%). In the case of wells containing photoactivator, the degree of reduction was calculated by referring to the number of CFU (colony-forming units) obtained from the cultivation of the positive control.

The data obtained in relation to CFU/mL were logarithmically transformed. In order to identify the differences between the groups within the transformed data, the Kruskal–Wallis one-directional nonparametric analysis of variance was performed. A U–Mann–Whitney test was applied to compare the differences between any two groups. Statistical comparisons were performed using SPSS software version 19 (SPSS, Chicago, IL, USA). For all analyses, *p* < 0.05 was considered statistically significant.

## 3. Results

In the case of wells not treated with laser or photodisinfection, the degree of growth reduction was insignificant, the variations being less than 10% compared to the positive control in the case of all active substances and their combinations (Table 2):

In the case of laser application, the reduction in microbial multiplication was evident, with variations depending on the treatment method (Table 3):

## 4. Discussion

There is a growing awareness in the scientific community of the effect of the diversity and composition of the microbiota on a patient’s response to synthetic and biological immunosuppressive therapy. Therapies such as cyclophosphamide and methotrexate (MTX) can induce a diffuse depletion of the intestinal microbiota, associated with the increase in commensal species in favor of pathogens that can damage the intestinal barrier, changing the permeability of epithelial cells, with consequent bacterial translocation. The risk of infections in patients with autoimmune diseases is higher than in healthy subjects, due to both endogenous factors (dysfunctional immune system) and exogenous factors (comorbidities and immunosuppressive therapy). Moreover, infections can induce relapses or cause a more severe clinical evolution, sometimes causing death in immunocompromised subjects [18]. Immunosuppressive treatment is the main exogenous factor contributing to the increased risk of infections in such patients. Anti-TNFα agents are an approved therapeutic line in the treatment of autoimmune diseases and, although these agents have demonstrated safety and a good efficacy profile, some patients may experience adverse effects related to treatment administration [19].

In our study, the growth of *A. actinomycetemcomitans* in vitro in the absence of any therapy was slightly inhibited by the drug combinations Etanercept 2.5 μg/mL + Methotrexate 2.5 μg/mL, and Infliximab 50 μg/mL + Methotrexate 2.5 μg/mL. In the absence of other studies in the literature that analyze in vitro the effect of these immunomodulating drugs on a strain of *A. actinomycetemcomitans*, we can assume that they act by inhibiting certain virulence factors that are expressed at the level of bacterial multiplication. Because anti-TNF-α drug treatment is commonly used to control the inflammatory process, such therapy may also be relevant for the management of periodontitis. Some data in the literature suggest that epigenetic changes through regulating pro-inflammatory responses via NFkB affecting TNF-α may be significantly involved in the pathogenesis of rheumatoid arthritis and other chronic inflammatory diseases [20]. Taking into account the data from microbiological, immunological and histopathological studies that indicate that *A. actinomycetemcomitans* is important in the etiology of periodontal disease, being a potentially virulent bacterium including multiple immune evasion mechanisms, each with a crucial role in the conversion of the periodontal tissue to a pathological status, we can also take into account the hypothesis of the modulation by the pharmaceutical combinations used in this study and of the profoundly altered oxidative stress autoimmune diseases. 

*P. gingivalis* and *A. actinomycetemcomitans* were observed to induce IL-17 production by CD4+ T cells when cultured with monocytes. *A. actinomycetemcomitans* induced IL-17 production and monocyte activation in a lower number when compared to *P. gingivalis*. The ability of cells stimulated with these periodontopathogens to produce IL-6, IL-1β, TNF-α and IL-23 may contribute to the induction of Th17 in situ. These authors concluded that *P. gingivalis* and *A. actinomycetemcomitans* can activate monocytes, the result being increased IL-17 production by CD4+ T cells in vitro [21].

Photodynamic therapy and diode laser therapy are used as an adjunct to conventional periodontal therapy, not as replacements for it. These methods must be implemented after the mechanical disaggregation of the biofilm has been achieved, targeting the floating form of bacteria in the periodontal pocket. Although in vivo periodontal pathogens live in complex communities, either in the form of a biofilm or in a floating form, we chose an experimental model of bacterial monoculture due to the fact that it allows the investigation of variables under single, standardized, reproducible test conditions. This was also the reason why we chose to optimize within our in vitro experimental study the bacterial monocultures of *Aggregatibacter actinomycetemcomitans* in a planktonic state.

We evaluated the effect of laser and photodynamic therapies in rheumatoid arthritis patients in a previous study. Clinical periodontal measurements and oxidative stress markers (8-hydroxy-2′-deoxyguanosine (8-OHdG) and 4 hydroxynonenal (4-HNE)) were analyzed at baseline and 6 months after periodontal treatment and we observed that the analyzed oxidative stress markers decreased significantly following non-surgical periodontal therapy in both rheumatoid arthritis and systemically healthy patients. Moreover, the association of laser therapy with scaling and root planing and photodisinfection with scaling and root planing yielded the best clinical and paraclinical outcomes when compared to classical periodontal therapy alone [22].

Ultraviolet (UV) light has been used as phototherapy for various skin diseases, such as psoriasis, atopic dermatitis, vitiligo and photodermatoses. The wavelengths of UV light are classified as UVC (100–280 nm), UVB (280–315 nm) and UVA (315–400 nm). UVA and UVB radiation have often been used to treat dermatoses. UVB light irradiation has been reported to alter T regulatory (Treg) cells and exhibit immunosuppressive effects in patients with psoriasis and atopic dermatitis [23]. Therefore, UVB is effective in treating various skin diseases mediated by immunomodulation and could also be useful for the treatment of oral alterations such as periodontal disease. Takada et al. demonstrated in 2017 that irradiation with 310 nm UV-LED radiation had weak bactericidal effects on oral bacteria, but showed low toxicity on gingival epithelial cells; moreover, irradiation induced the production of ROS and was particularly harmful to *P. gingivalis*. These results suggest that the 310 nm UV-LED irradiation device may be useful for the treatment of periodontal disease, but further research is needed to evaluate the benefits and precise indications of this therapy [24].

It has been shown that 310 nm UVB irradiation induces an immunosuppressive reaction in the skin, causing an increase in IL-10 production, a decrease in the number of Th1 cells and an increase in Th2 cells [25]. Immune hyperreactivity has been associated with the pathogenesis of chronic periodontal disease and the relationships between these cytokines, T cells and periodontal disease has been reported [26]. Thus, the immunosuppressive reaction induced by UVB irradiation can be effective in the treatment of chronic periodontal disease.

UVC radiation has the strongest bactericidal effects and, in particular, around 254 nm, UVC is mainly absorbed in the DNA. UVC-LED radiation of 265 nm has a stronger bactericidal effect than UVB-LED irradiation. UVB irradiation also shows DNA damage, with the generation of pyrimidine dimers, but the effect is negligible. Therefore, UVB irradiation induces only low toxicity on gingival epithelial cells. Periodontal pathogenic bacteria such as *P. gingivalis* are anaerobic bacteria and are sensitive to reactive oxygen species; conversely, other oral resident bacteria, such as streptococci, are tolerant to reactive oxygen species [24]. Therefore, reactive oxygen species induced by irradiation with a UVB-LED can selectively annihilate periodontopathogenic bacteria and cause the oral bacterial flora to change from periodontopathogenic to non-periodontopathogenic.

Blue LED light sources are commonly used in dentistry for the photopolymerization of dental resins. *A. actinomycetemcomitans* was inactivated at a rate exceeding five log10 steps of CFU after a 120 s irradiation with blue light derived from a dental unit (LED); however, when reducing the irradiation time to 20 and 40 s, the inactivation of the microbiological agent was ≤1 and 2 log10 steps, respectively. An effect due to illumination-induced heat in the samples can be neglected, as the temperature increase was only marginal (about 3 °C after illumination for 120 s). The blue light had no bactericidal effect on *E. coli*, which was chosen as a control because it has similar properties to *A. actinomycetemcomitans* and because its use as a negative control was already demonstrated in blue light phototoxicity experiments [27].

Photodisinfection therapy was tested by Oruba et al. in 2017 in an in vitro study that analyzed the effectiveness of this method using various operating parameters for the eradication of periodontopathogens. The conclusions of the study were that bacteria differ in their susceptibility to photodynamic inactivation, namely, an eradication of *P. gingivalis* and an inhibition of *F. nucleatum* was obtained, but the effect was not the same for *A. actinomycetemcomitans*, which proved to be insensitive to such therapeutic applications [28].

In our study, a degree of reduction >99.9 (3log) was obtained for photodisinfection therapy with a photosensitizer based on methylene blue, after a 30 s exposure to light radiation, regardless of the drug combinations used.

Another study investigated the effect of methylene blue-mediated antimicrobial photodynamic therapy (PDT) on cell viability and expression of the fimbriae-associated gene (rcpA) in *A. actinomycetemcomitans*. To determine the dose-dependent effects of PDT, a strain of *A. actinomycetemcomitans* (ATCC 33384) was irradiated after exposure to methylene blue, followed by cell survival analysis and expression ratio of rcpA by CFU and real-time PCR testing. In that study, the administration of 100 μg/mL methylene blue caused a significant reduction in the growth of *A. actinomycetemcomitans* compared to the control (*p* < 0.05). The sub-lethal dose of PDT against *A. actinomycetemcomitans* was 25 µg/mL at a fluence of 93.75 J/cm^2^. The sub-lethal dose of PDT could lead to an approximately fourfold suppression of rcpA expression, thus significantly reducing the expression of rcpA as an important virulence factor of this strain in cells, and, therefore, PDT may be a valuable tool for the treatment of *A. actinomycetemcomitans* infections [29].

Fekrazad et al., exposed cultures of *A. actinomycetemcomitans* to a 662 nm laser in the presence of the photosensitizer Radachlorin^®^ and to an 810 nm laser in the presence of the photosensitizer EmunDo^®^, then the bacterial suspension of each well in the study groups was diluted and subcultured on the surface of Muller–Hinton agar plates, with CFU analysis. *A. actinomycetemcomitans* suspensions showed a significant reduction in the case of both therapies, being thus recommended by the authors as a promising new approach in neutralizing periodonto-pathogenic bacteria [30].

Another study, this time performed on biofilm, concluded that, separately, a photoactivator based on methylene blue and light radiation cannot induce the inactivation of *A. actinomycetemcomitans*. The authors also observed that photodisinfection is dependent on exposure time, with the highest bacterial reduction (99.85%, *p* = 0.0004) occurring after exposure to methylene blue and photodynamic for 5 min, and for these parameters, the biofilm also suffered important structural damage [31].

The efforts to find new photosensitizing agents are evident in the literature, with two in vitro studies from 2018 proposing a curcuma extract with a 450 nm light source to be used for this purpose. Both studies concluded that the toxic effect on *A. actinomycetemcomitans* was dose-dependent and that this substance could be used as a photosensitizing agent in periodontal disease therapy [32,33].

Zirconia implants were contaminated with a bacterial suspension of *Prevotella intermedia, Actinomyces actinomycetemcomitans* and *Porphyromonas gingivalis* and then randomly assigned to four groups according to the decontamination protocol: Group 1 (PDT1)-PDT (660 nm, 100 mW) with toluidine blue; Group 2 (PDT2)-PDT (660 nm, 100 mW) with phenothiazine chloride; Group 3 (LAD)–LED device with toluidine blue; and Group 4 (TB)-toluidine blue without the application of light. The analysis recorded in all study groups significant reductions in the number of CFU compared to controls (*p* < 0.05) and PDT1, PDT2 and LAD had the highest bacterial reduction with respect to each separate bacterial species but also with respect to the total number of bacteria. The SEM analysis of the implant surfaces did not show any changes after the treatment procedures, thus demonstrating high effectiveness in the decontamination of zirconium dental implants [34]. Thus, the indications of photodynamic therapy could also be extended in the treatment of peri-implantitis, a pathology that is difficult to treat and which has serious consequences on the oral cavity.

For the diode laser therapy, the most significant reductions in the amount of *A. actinomycetemcomitans* were obtained when irradiating with a power of 5 W, the percentages being >98% regardless of the pharmaceutical associations. The laser we use has a wavelength of 940 nm, which is in the infrared spectrum. We chose this wavelength because in this particular interval, there is a maximum absorption of melanin, hemoglobin and water, compared to other parts of the infrared spectrum, so we can obtain a maximum antimicrobial effect but at the same time a protection of the host cells.

A study observed that the density of power did not modify the photosensitizers’ absorption of light. *A. actinomycetemcomitans* was inactivated completely through the association of proper photosensitizer absorption and irradiation characteristics [35]. 

A systematic review analyzed a total of 32 in vitro studies, among those, 25 used in-suspension microorganisms and observed a reduction greater than or equal to 3 logs CFU/mL of periodontopathogens. In biofilms, three studies highlighted showed a reduction equal to or greater than 3 logs CFU/mL. Nonetheless, the authors stress the importance of light parameters standardization, photosensitizer type and pre-irradiation time prior to performing clinical studies [36]. 

However, other authors stress the inefficacy of one session of photodisinfection, concluding that solely one application of this technique in adjunction to non-surgical periodontal treatment is inefficient in *P. gingivalis* and *A. actinomycetemcomitans* positive periodontitis patients [37].

Recent studies have tested biofilms of *A. actinomycetemcomitans* plus *Streptococcus sanguinis* grown on bovine root surfaces, treated with an 810 nm diode laser, pulsed mode, non-contact, with an interval and pulse length of 20 ms. Four protocols were tested, namely, one episode of 1.5 or 2.5 W for 30 s and three episodes of 1.5 or 2.5 W for 30 s. The authors used as negative control the absence of any treatment and 0.2% chlorhexidine as positive control. Chlorhexidine treatment and all laser protocols except for that using a single episode of 1.5 W reduced the number of *A. actinomycetemcomitans* in either the single-species or the dual-species biofilm compared to the negative control. The authors concluded that a higher percentage of *A. actinomycetemcomitans* reduction occurred after increasing the power or repeating the irradiation, but they failed to eradicate the biofilm regardless of the applied protocol [16].

Analysis of the antimicrobial efficiency of irradiation with light from the visible spectrum on oral bacteria in vitro, through the evaluation of an impressive number of studies that took into account various oral pathogens (incriminated particularly in periodontitis) such as *Fusobacterium* spp., *Porphyromonas* spp., *Aggregatibacter* spp., *Prevotella* spp., *Staphylococcus* spp., *Streptococcus* spp., showed that laser irradiation could be a viable option for controlling *A. actinomycetemcommitans* infection, but for best results light sources with the absorption spectrum for flavins and porphyrins should be used; practically, it appears that the eradication of bacteria in planktonic cultures is especially effective in the case of black-pigmented ones, such as *Porphyromonas* and *Prevotella* spp. Regarding bacteria organized in biofilms, the reported evidence is less clear, and more studies that take into account multiple working protocols are needed [7].

A study found that *A. actinomycetemcomitans* initiates neutrophil-mediated leukotoxin A (LtxA) hypercitrullination, being detected in the oral microbiome of rheumatoid arthritis patients, where it could act as a bacterial trigger of the disease [38]. The development of therapeutic strategies targeting this bacterium could prevent the onset, or improve the course of, rheumatoid arthritis developed through this etiopathogenic pathway.

Overall, the analyzed results imply that laser and photodynamic therapy could be a treatment option for the eradication of *A. actinomycetemcommitans*. However, additional studies are needed, especially regarding the existence of the biofilm, as very likely *A. actinomycetemcomitans* is not the only microorganism capable of inducing hypercitrullination in neutrophils.

Photodisinfection and diode laser therapy could be an effective option for antimicrobial applications in dentistry, for example, in the treatment of periodontal disease and peri-implantitis, given that blue light irradiation has also been shown to be effective against other periodontal pathogens such as *Porphyromonas gingivalis*, *F. nucleatum* and *Prevotella* spp. However, further studies are necessary [39,40,41].

Limitations of this study consist of the inherent limitations of an in vitro study, in that the conclusions need to be extrapolated with care to the clinical implications. In this study, we only analyzed the effectiveness of a diode laser and photodisinfection on a single bacterial strain of *A. actinomycetemcommitans*; however, the oral environment is much more complex. 

In future studies, we will expand our research to other immunomodulatory medications and their effect on other periodonto-pathogens. 

## 5. Conclusions

The association of a conventional antirheumatic drug with anti-TNF-α therapy determined a significantly greater inhibition of a type strain of *A. actinomycetemcomitans* compared to monotherapy, in vitro.

Photodisinfection caused a significant reduction in a type strain of *A. actinomycetemcomitans* after a 30 s exposure in vitro, regardless of the pharmaceutical associations of biological and conventional DMARDs.

Irradiation with a diode laser for 30 s at a power of 5 W caused a greater reduction in *A. actinomycetemcomitans* compared to irradiation with 1 W.

Our in vitro study did not record statistically significant differences between the eradication of *A. actinomycetemcomitans* by photodynamic therapy with methylene blue and the diode laser at a power of 5 W.

Future studies should focus on identifying possible interactions between the applied methods in order to implement them successfully in standardized and optimized clinical protocols.

## Figures and Tables

**Table 1:** Studies that analyze the effect of laser and photodisinfection therapy on periopathogenic bacteria in vitro.

Technique	Bacteria	Effect	Author
PDT + toluidine or methylene blue dye	*A. actinomycetemcomitans*	100% eradication at 10 mg/mL	Valle et al., 2019 [14]
PDT + rose bengal	*P. gingivalis, A. actinomycetemcomitans, F. nucleatum*	Maximal reduction at 160 μg/mL rose bengal	Wang et al., 2021 [15]
Diode Laser 810-nm	*A. actinomycetemcomitans*	93% reduction at 2.5 W; 30 s	Tantivitayakul et al., 2018 [16]
Diode laser 635 nm + phycocyanin	*P. gingivalis*	Mean reduction 44.24%	Etemadi et al., 2022 [17]

**Table 2:** Values of *A. actinomycetemcomitans* for the wells not submitted to adjuvant therapy.

Active Substance	E	E + M	I	I + M	M	CP	CN
Absorbance value (mean and standard deviation)	2.054 ± 0.197	1.922 ± 0.189	2.031 ± 0.214	1.968 ± 0.191	2.075 ± 0.174	2.163 ± 0.209	0.154 ± 0.028

Etanercept 2.5 μg/mL (E); Infliximab 50 μg/mL (I); Metotrexat 2.5 μg/mL (M); Etanercept 2.5 μg/mL + Metotrexat 2.5 μg/mL (E + M); Infliximab 50 μg/mL + Metotrexat 2.5 μg/mL (I + M).

**Table 3:** Values of *A. actinomycetemcomitans* for the wells submitted to adjuvant laser therapy and photodynamic therapy.

Active Substance	E	E + M	I	I + M	M	CP	CN
Absorbance value (mean and standard deviation)	0.402 ± 0.0	0.384 ± 0.051	0.358 ± 0.067	0.353 ± 0.046	0.398 ± 0.051	2.163 ± 0.209	0.154 ± 0.028
Degree of reduction (%)	87.7	88.6	90.1	90.1	87.9	0.00	-
Absorbance value (mean and standard deviation) 5W	0.176 ± 0.052	0.172 ± 0.048	0.168 ± 0.051	0.161 ± 0.039	0.189 ± 0.044	2.163 ± 0.209	0.154 ± 0.028
Degree of reduction %)	98.9	99.1	99.3	99.7	98.3	0.00	-
CFU/mL resulting from cultivation (mean)	<102	<102	<102	<102	<102	2.6 ± 105	-
Degree of reduction % (log10)	>99.9 (3log)	>99.9 (3log)	>99.9 (3log)	>99.9 (3log)	>99.9 (3log)	0.00	-

Etanercept 2.5 μg/mL (E); Infliximab 50 μg/mL (I); Metotrexat 2.5 μg/mL (M); Etanercept 2.5 μg/mL + Metotrexat 2.5 μg/mL (E + M); Infliximab 50 μg/mL + Metotrexat 2.5 μg/mL (I + M).

## Data Availability

All data are available from the corresponding authors upon reasonable request.

## References

[1] Kapila Y.L. (2021). Oral health’s inextricable connection to systemic health: Special populations bring to bear multimodal relationships and factors connecting periodontal disease to systemic diseases and conditions. Periodontology 2000.

[2] González-Febles J., Sanz M. (2021). Periodontitis and rheumatoid arthritis: What have we learned about their connection and their treatment?. Periodontology 2000.

[3] Zhao H., Hu J., Zhao L. (2021). The effect of low-level laser therapy as an adjunct to periodontal surgery in the management of postoperative pain and wound healing: A systematic review and meta-analysis. Lasers Med. Sci..

[4] Isola G., Polizzi A., Patini R., Ferlito S., Alibrandi A., Palazzo G. (2020). Association among serum and salivary *A. actinomycetemcomitans* specific immunoglobulin antibodies and periodontitis. BMC Oral Health.

[5] Ardila C.M., Bedoya-García J.A., Arrubla-Escobar D.E. (2022). Antibiotic resistance in periodontitis patients: A systematic scoping review of randomized clinical trials. Oral Dis..

[6] Akram Z., Hyder T., Al-Hamoudi N., Binshabaib M.S., Alharthi S.S., Hanif A. (2017). Efficacy of photodynamic therapy versus antibiotics as an adjunct to scaling and root planing in the treatment of periodontitis: A systematic review and meta-analysis. Photodiagnosis Photodyn. Ther..

[7] Pummer A., Knüttel H., Hiller K.A., Buchalla W., Cieplik F., Maisch T. (2017). Antimicrobial efficacy of irradiation with visible light on oral bacteria in vitro: A systematic review. Future Med. Chem..

[8] Mocanu R.C., Martu M.-A., Luchian I., Sufaru I.G., Maftei G.A., Ioanid N., Martu S., Tatarciuc M. (2021). Microbiologic Profiles of Patients with Dental Prosthetic Treatment and Periodontitis before and after Photoactivation Therapy—Randomized Clinical Trial. Microorganisms.

[9] Sculean A., Deppe H., Miron R., Schwarz F., Romanos G., Cosgarea R. (2021). Effectiveness of photodynamic therapy in the treatment of periodontal and peri-implant diseases. Oral Biofilms.

[10] Nicolae V., Chiscop I., Cioranu V.S.I., Martu M.A., Luchian A.I., Martu S., Solomon S.M. (2015). The use of photoactivated blue-o toluidine for periimplantitis treatment in patients with periodontal disease. Rev. Chim..

[11] Rola P., Włodarczak S., Lesiak M., Doroszko A., Włodarczak A. (2022). Changes in Cell Biology under the Influence of Low-Level Laser Therapy. Photonics.

[12] Colaco A.S. (2018). An update on the effect of low-level laser therapy on growth factors involved in oral healing. J. Dent. Lasers.

[13] Ren C., McGrath C., Jin L., Zhang C., Yang Y. (2017). The effectiveness of low-level laser therapy as an adjunct to non-surgical periodontal treatment: A meta-analysis. J. Periodontal Res..

[14] Valle L.A., Lopes M.M., Zangrando M.S., Sant’Ana A.C., Greghi S.L., de Rezende M.L., Damante C.A. (2019). Blue photosensitizers for aPDT eliminate *Aggregatibacter actinomycetemcomitans* in the absence of light: An in vitro study. J. Photochem. Photobiol. B Biol..

[15] Wang D., Pan H., Yan Y., Zhang F. (2021). Rose bengal-mediated photodynamic inactivation against periodontopathogens in vitro. Photodiagnosis Photodyn. Ther..

[16] Tantivitayakul P., Rassameemasmaung S., Thapanabhiboonsuk S. (2018). In vitro effect of diode laser against biofilm of Aggregatibacter actinomycetemcomitans. Eur. J. Dentistry..

[17] Etemadi A., Azizi A., Pourhajibagher M., Chiniforush N. (2022). In Vitro Efficacy of Antimicrobial Photodynamic Therapy with Phycocyanin and Diode Laser for the Reduction of *Porphyromonas gingivalis*. J. Lasers Med. Sci..

[18] Picchianti-Diamanti A., Rosado M.M., D’Amelio R. (2018). Infectious agents and inflammation: The role of microbiota in autoimmune arthritis. Front. Microbiol..

[19] Wysocki T., Paradowska-Gorycka A. (2022). Pharmacogenomics of Anti-TNF Treatment Response Marks a New Era of Tailored Rheumatoid Arthritis Therapy. Int. J. Mol. Sci..

[20] Zamri F., De Vries T.J. (2020). Use of TNF inhibitors in rheumatoid arthritis and implications for the periodontal status: For the benefit of both?. Front. Immunol..

[21] Cheng W.C., van Asten S.D., Burns L.A., Evans H.G., Walter G.J., Hashim A., Hughes F.J., Taams L.S. (2016). Periodontitis-associated pathogens *P. gingivalis* and *A. actinomycetemcomitans* activate human CD14+ monocytes leading to enhanced Th17/IL-17 responses. Eur. J. Immunol..

[22] Martu M.-A., Surlin P., Lazar L., Maftei G.A., Luchian I., Gheorghe D.-N., Rezus E., Toma V., Foia L.-G. (2021). Evaluation of Oxidative Stress before and after Using Laser and Photoactivation Therapy as Adjuvant of Non-Surgical Periodontal Treatment in Patients with Rheumatoid Arthritis. Antioxidants.

[23] Ibbotson S.H. (2018). A perspective on the use of NB-UVB phototherapy versus PUVA photochemotherapy. Front. Med..

[24] Takada A., Matsushita K., Horioka S., Furuichi Y., Sumi Y. (2017). Bactericidal effects of 310 nm ultraviolet light-emitting diode irradiation on oral bacteria. BMC Oral Health.

[25] Elnazar A., Salma Y., Ghazy A.A., Ghoneim H.E., Taha A.R., Abouelella A.M. (2015). Effect of ultra violet irradiation on the interplay between Th1 and Th2 lymphocytes. Front. Pharmacol..

[26] Ebersole J.L., Dawson D., Emecen-Huja P., Nagarajan R., Howard K., Grady M.E., Thompson K., Peyyala R., Al-Attar A., Lethbridge K. (2017). The periodontal war: Microbes and immunity. Periodontology 2000.

[27] Cieplik F., Späth A., Leibl C., Gollmer A., Regensburger J., Tabenski L., Hiller K.A., Maisch T., Schmalz G. (2014). Blue light kills *Aggregatibacter actinomycetemcomitans* due to its endogenous photosensitizers. Clin. Oral Investig..

[28] Oruba Z., Łabuz P., Macyk W., Chomyszyn-Gajewska M. (2017). Periopathogens differ in terms of the susceptibility to toluidine blue O-mediated photodynamic inactivation. Photodiagnosis Photodyn. Ther..

[29] Pourhajibagher M., Monzavi A., Chiniforush N., Monzavi M.M., Sobhani S., Shahabi S., Bahador A. (2017). Real-time quantitative reverse transcription-PCR analysis of expression stability of *Aggregatibacter actinomycetemcomitans* fimbria-associated gene in response to photodynamic therapy. Photodiagnosis Photodyn. Ther..

[30] Fekrazad R., Khoei F., Bahador A., Hakimiha N. (2017). Photo-activated elimination of *Aggregatibacter actinomycetemcomitans* in planktonic culture: Comparison of photodynamic therapy versus photothermal therapy method. Photodiagnosis Photodyn. Ther..

[31] Alvarenga L.H., Prates R.A., Yoshimura T.M., Kato I.T., Suzuki L.C., Ribeiro M.S., Ferreira L.R., dos Santos Pereira S.A., Martinez E.F., Saba-Chujfi E. (2015). *Aggregatibacter actinomycetemcomitans* biofilm can be inactivated by methylene blue-mediated photodynamic therapy. Photodiagnosis Photodyn. Ther..

[32] Saitawee D., Teerakapong A., Morales N.P., Jitprasertwong P., Hormdee D. (2018). Photodynamic therapy of Curcuma longa extract stimulated with blue light against *Aggregatibacter actinomycetemcomitans*. Photodiagnosis Photodyn. Ther..

[33] Pourhajibagher M., Chiniforush N., Monzavi A., Barikani H., Monzavi M.M., Sobhani S., Shahabi S., Bahador A. (2018). Inhibitory Effects of Antimicrobial Photodynamic Therapy with Curcumin on Biofilm-Associated Gene Expression Profile of *Aggregatibacter actinomycetemcomitans*. J. Dent..

[34] Azizi B., Budimir A., Bago I., Mehmeti B., Jakovljević S., Kelmendi J., Stanko A.P., Gabrić D. (2018). Antimicrobial efficacy of photodynamic therapy and light-activated disinfection on contaminated zirconia implants: An in vitro study. Photodiagnosis Photodyn. Ther..

[35] de Sousa G.R., Soares L.O., Soares B.M., de Carvalho Cruz R., Uliana Junior P., Santiago T., Farias L.M., Magalhães P.P., Silveira L.B., Almeida Lopes L. (2022). In vitro evaluation of physical and chemical parameters involved in aPDT of *Aggregatibacter actinomycetemcomitans*. Lasers Med. Sci..

[36] Sales L.S., Miranda M.L., de Oliveira A.B., Ferrisse T.M., Fontana C.R., Milward M., Brighenti F.L. (2022). Effect of the technique of photodynamic therapy against the main microorganisms responsible for periodontitis: A systematic review of in-vitro studies. Arch. Oral Biol..

[37] Aabed K., Moubayed N., BinShabaib M.S., ALHarthi S.S. (2022). Is a single session of antimicrobial photodynamic therapy as an adjuvant to non-surgical scaling and root planing effective in reducing periodontal inflammation and subgingival presence of Porphyromonas gingivalis and *Aggregatibacter actinomycetemcomitans* in patients with periodontitis?. Photodiagnosis Photodyn. Ther..

[38] Konig M.F., Abusleme L., Reinholdt J., Palmer R.J., Teles R.P., Sampson K., Rosen A., Nigrovic P.A., Sokolove J., Giles J.T. (2016). *Aggregatibacter actinomycetemcomitans*–induced hypercitrullination links periodontal infection to autoimmunity in rheumatoid arthritis. Sci. Transl. Med..

[39] Salvi G.E., Stähli A., Schmidt J.C., Ramseier C.A., Sculean A., Walter C. (2020). Adjunctive laser or antimicrobial photodynamic therapy to non-surgical mechanical instrumentation in patients with untreated periodontitis: A systematic review and meta-analysis. J. Clin. Periodontol..

[40] Choe R., Balhaddad A.A., Fisher J.P., Melo M.A., Huang H.C. (2021). Photodynamic Therapy for Biomodulation and Disinfection in Implant Dentistry: Is It Feasible and Effective?. Photochem. Photobiol..

[41] Sufaru I.-G., Martu M.-A., Luchian I., Stoleriu S., Diaconu-Popa D., Martu C., Teslaru S., Pasarin L., Solomon S.M. (2022). The Effects of 810 nm Diode Laser and Indocyanine Green on Periodontal Parameters and HbA1c in Patients with Periodontitis and Type II Diabetes Mellitus: A Randomized Controlled Study. Diagnostics.

